# A novel selenium analog of HDACi-based twin drug induces apoptosis and cell cycle arrest via CDC25A to improve prostate cancer therapy

**DOI:** 10.7150/thno.92119

**Published:** 2024-06-03

**Authors:** Zhiyong Shi, Miaomiao Liu, Xiaowen Zhang, Jingyang Wang, Junwei Zhang, Zeyan Peng, Li Meng, Ruijing Wang, Lihong Guo, Qiang Zhang, Jing Li, Liang Yang, Jie Liu, Yang Xu, Jie Yan, Jianlin Cui, Shan Ren, Yang Gao, Yanming Wang, Zhi Qi

**Affiliations:** 1 Department of Molecular Pharmacology, School of Medicine; College of Pharmacy, Tianjin Key Laboratory of Molecular Drug Research, Nankai University, Tianjin 300350, China.; 2 Institute of Digestive Disease, Shengli Oilfield Central Hospital, Dongying 257000, China.; 3 Department of Clinical Laboratory, Branch of Tianjin Third Central Hospital, Tianjin 300250, China.; 4 The First Department of Critical Care Medicine, The First Affiliated Hospital of Shihezi University, Shihezi, 832003, China.; 5 Tianjin Key Laboratory of General Surgery in Construction, Tianjin Union Medical Center, Tianjin 300122, China.

**Keywords:** HDAC inhibitors, SAHA selenium analogs, synergistic therapy, PDH, CDC25A

## Abstract

Cancer therapy has moved from single agents to more mechanism-based targeted approaches. In recent years, the combination of HDAC inhibitors and other anticancer chemicals has produced exciting progress in cancer treatment. Herein, we developed a novel prodrug via the ligation of dichloroacetate to selenium-containing potent HDAC inhibitors. The effect and mechanism of this compound in the treatment of prostate cancer were also studied.

**Methods:** The concerned prodrug SeSA-DCA was designed and synthesized under mild conditions. This compound's preclinical studies, including the pharmacokinetics, cell toxicity, and anti-tumor effect on prostate cancer cell lines, were thoroughly investigated, and its possible synergistic mechanism was also explored and discussed.

**Results:** SeSA-DCA showed good stability in physiological conditions and could be rapidly decomposed into DCA and selenium analog of SAHA (SeSAHA) in the tumor microenvironment. CCK-8 experiments identified that SeSA-DCA could effectively inhibit the proliferation of a variety of tumor cell lines, especially in prostate cancer. In further studies, we found that SeSA-DCA could also inhibit the metastasis of prostate cancer cell lines and promote cell apoptosis. At the animal level, oral administration of SeSA-DCA led to significant tumor regression without obvious toxicity. Moreover, as a bimolecular coupling compound, SeSA-DCA exhibited vastly superior efficacy than the mixture with equimolar SeSAHA and DCA both *in vitro* and *in vivo*. Our findings provide an important theoretical basis for clinical prostate cancer treatment.

**Conclusions:** Our *in vivo* and *in vitro* results showed that SeSA-DCA is a highly effective anti-tumor compound for PCa. It can effectively induce cell cycle arrest and growth suppression and inhibit the migration and metastasis of PCa cell lines compared with monotherapy. SeSA-DCA's ability to decrease the growth of xenografts is a little better than that of docetaxel without any apparent signs of toxicity. Our findings provide an important theoretical basis for clinical prostate cancer treatment.

## Introduction

Prostate cancer (PCa) ranks second as the most prevalent cancer and fifth among the leading causes of death in males 1. At present, a range of extensive therapies centered on androgen deprivation therapy can be utilized. However, after 18-24 months of treatment, castration-resistant prostate cancer (CRPC) can progress with a poor prognosis in most patients 2. Chemotherapy based on cytotoxic drugs remains among the most potent therapies for patients diagnosed with CRPC. Although the combination of docetaxel and prednisone can increase the overall survival of patients with metastatic PCa and has been favored as a treatment for those with metastatic CRPC 3, a subset of patients eventually become resistant to treatment due to multidrug resistance, decreased drug sensitivity, and severe side effects. Consequently, the pursuit of new anti-cancer drugs or different combinations of medication has gained popularity in the management of PCa.

Histone deacetylases (HDACs) have become significant targets for pharmaceutical intervention in various diseases due to their role in controlling chromatin remodeling. They have emerged as important pharmaceutical targets for a wide range of diseases because they regulate chromatin remodeling 4, gene transcription 5, cell proliferation 6, and apoptosis 7. Research has indicated that the incidence of class I HDACs expression, such as HDAC1 and HDAC2, escalates in PCa and is more widespread in individuals with CRPC, adversely affecting the disease's prognosis to a significant extent 8. The FDA has already approved various forms of HDAC inhibitors for the treatment of hematological malignancies 9, 10. For example, suberoylanilide hydroxamic acid (SAHA), a pan-HDAC inhibitor, also demonstrates significant potential as an emerging medication for treating solid tumors 11, 12. However, its efficacy in the treatment of PCa remains unsatisfactory. The main challenges are limited oral bioavailability, poor stability, and lack of solid tumor uptake.

Dichloroacetate (DCA) regulates the function of pyruvate dehydrogenase (PDH) and enhances the entry of pyruvate produced during glycolysis into the mitochondria, where the tricarboxylic acid cycle takes place, by inhibiting the activity of PDH kinase 13, 14. Studies have demonstrated that it possesses anti-cancer characteristics by triggering cell-cycle arrest and apoptosis in tumor cells 15. In addition, DCA can provide sustained drug release when it binds to a larger structure, such as amide derivatives 13.

In previous studies, we synthesized a newly developed selenium analog of SAHA (SeSAHA) and compared its inhibitory activity against SAHA. The results showed that this selenium-containing compound had a greater inhibitory effect (more than 10-fold) than the known inhibitor (data not shown). Based on this research, we used simple esterification to ligate the DCA molecule to the SeSAHA molecule to form a novel prodrug called suberoylanilide hydroxamic acid-dichloroacetate (SeSA-DCA). By concurrently reducing glutathione (GSH) and increasing reactive oxygen species (ROS), SeSA-DCA can effectively inhibit the activity of HDAC, p-PDH, and CDC25A both *in vitro* and *in vivo*, blocking PCa cell lines in the G1 phase, and induce cell apoptosis, thus exhibiting a promising anti-cancer effect both *in vitro* and *in vivo*. Surprisingly, the IC_50_ value of its treatment of PCa was 10^2^ times lower than that of SAHA, and it also showed a better suppression ability of tumor growth than docetaxel *in vivo*. This study also exhibits a successful application of the hybrid molecule approach for developing effective anti-cancer compounds.

## Methods

### Materials and instrumentation

Se powder was purchased from Titan Technology Co., LTD (Shanghai, China). Sodium borohydride (NaBH_4_), tetrahydrofuran (THF), sodium hydroxide (NaOH), 1,4-Dioxane, N, N-dimethylformamide (DMF), triethylamine (Et3N), and hydrogen peroxide (H_2_O_2_) were purchased from Bohua Chemical Reagent Co., LTD (Tianjin, China). Ethyl 7-bromoheptanoate was obtained from Bidde Medical Technology Co. LTD (Shanghai, China). Three-amino benzyl alcohol and 1-Hydroxy-7-azabenzotriazole were purchased from Sinoop Technology Co. LTD (Tianjin, China). Two- (7-Azabenzotriazol-1-yl)-N, N, N', N'-tetramethyluronium hexafluorophosphate (HATU) and N, N-Diisopropylethylamine were obtained from Aladdin (Shanghai, China). DCA was purchased from Enoch, LTD (Beijing, China). DTT was purchased from Myrell Chemical Technologies, LTD (Shanghai, China). Except for H_2_O_2_ and ethyl 7-bromoheptanoate, all the organic solvents were used after drying with a 4A molecular sieve. All other chemicals and biological reagents were used as received without further purification. ^1^H-NMR and ^77^Se-NMR spectra were recorded on a Bruker 400 M (400 MHz, Bruker, Karlsruhe, Germany) and an AV 400 (400 MHz, Bruker, Billerica, Massachusetts, USA) spectrometer, respectively. HPLC analysis was performed on an Alliance HPLC system (Waters China Co., Ltd., Beijing, China) equipped with an autosampler, variable UV wavelength detector, and analytical C18 column (InertSustain, 250 × 4.6 mm, 5 μm, GL Sciences Inc., Fukushima, Japan). ESI-MS and MALDI-MS were performed using a Fourier transform ion cyclotron resonance mass spectrometer (Varian 7.0T FTMS, San Carlos, California, USA) and a Fourier transform ion cyclotron resonance ultrahigh-resolution mass spectrometer (Solarix scimax MRMS, Bruker, Karlsruhe, Germany), respectively. The X-ray photoelectron spectroscopy was obtained by using an Axis ultra DLD multifunctional electronic spectrometer (Kratos Analytical Ltd., Manchester, UK).

### Synthesis of disodium diselenide (Na_2_Se_2_)

Selenium powder (0.2035 g, 2.577 mmol) and NaBH_4_ (0.195 g, 5.155 mmol) were first added to a three-necked round-bottom flask, followed by the addition of distilled water (5 mL) under a nitrogen atmosphere. The mixture was stirred in an ice bath. Then, another 2.5 mL distilled water was added by stirring into the flask and the mixture continued to react at RT for 1 h until a milky white aqueous solution was obtained. Finally, the rest of the selenium powder (0.2035 g, 2.577 mmol) was added and the solution was heated to 100 °C and refluxed for 2 h until a dark brownish-red solution was obtained, which was used directly in the next step.

### Synthesis of compound 1

Anhydrous THF (7.5 mL) was added to the dark brownish-red solution of Na_2_Se_2_ at 50 °C, and then ethyl 7-bromoheptanate (1.222 g, 5.155 mmol) was added dropwise and the mixture was stirred overnight. The THF was distilled under a vacuum. The residue was dissolved in ethyl acetate (50 mL) and washed with saturated NaCl solution (50 mL × 3). The ethyl acetate layer was dried over Na_2_SO_4_ and evaporated to give the crude product. The product was purified by silica gel column chromatography (petroleum ether/ethyl acetate = 40:1) to give compound 1 (0.7298 g, 60% yield) as a light yellow oil. ^1^H NMR (400 MHz, CDCl_3_): δ 4.10 (q, *J* = 7.1 Hz, 2H), 2.87 (t, *J* = 7.4 Hz, 2H), 2.27 (t, *J* = 7.5 Hz, 2H), 1.66 (dp, *J* = 39.9, 7.3 Hz, 4H), 1.45-1.30 (m, 4H), 1.23 (t, *J* = 7.1 Hz, 3H). ^13^C NMR (101 MHz, CDCl_3_): δ 60.18, 34.25, 30.75, 29.98, 29.12, 28.61, 24.82, 14.27.

### Synthesis of compound 2

Compound **1** (0.8171 g, 1.73 mmol) was dissolved in 1, 4-dioxane (7 mL) in a nitrogen atmosphere and stirred in an ice bath for 5 min. Thereafter, 3 M NaOH (7 mL) was added to the mixture and reacted at RT for 4 h. Then dichloromethane was used to extract the supernatant, and the pH value of the water phase was adjusted to 3.0 using 3 M HCl. The organic phase extracted with dichloromethane was washed thrice with saturated NaCl solution (80 mL × 3). The organic layer was dried over Na_2_SO_4_ and then filtered off. The dichloromethane was distilled under a vacuum to obtain compound **2** (0.6 g, 83.6% yield) as a light yellow solid. ^1^H NMR (400 MHz, CDCl_3_): δ 2.92 (p, *J* = 7.4 Hz, 2H), 2.37 (t, *J* = 7.1 Hz, 2H), 1.70 (dp, *J* = 29.6, 7.1 Hz, 4H), 1.47-1.32 (m, 4H). ^13^C NMR (101 MHz, CDCl_3_): δ 180.26, 34.01, 30.75, 30.02, 29.08, 28.48, 24.53.

### Synthesis of SeSAHA

To a solution of **2** (0.5 g, 1.201 mmol), 3-aminobenzyl alcohol (0.37 g, 3.003 mmol), HOAt (0.327 g, 2.402 mmol) and HATU (0.913 g, 2.402 mmol) in anhydrous DMF (10 mL) with nitrogen protection, DIEA (0.621 g, 4.804 mmol) was added at 0 °C. The reaction was stirred under a nitrogen atmosphere overnight at RT. Then, ethyl acetate (50 mL) was added and washed with saturated NaCl solution (50 mL × 2). The organic layer was dried over Na_2_SO_4_ and then filtered off. The ethyl acetate was distilled in a vacuum to obtain the crude product. The crude product was purified by recrystallization with ethyl acetate to obtain SeSAHA as a grey solid (0.725 g, 96% yield). ^1^H NMR (400 MHz, DMSO-*d_6_*): δ 9.82 (s, 1H), 7.55 (d, *J* = 1.9 Hz, 1H), 7.46 (dt, *J* = 8.1, 1.6 Hz, 1H), 7.21 (t, *J* = 7.8 Hz, 1H), 6.95 (d, *J* = 7.5 Hz, 1H), 5.17 (t, *J* = 5.7 Hz, 1H), 4.45 (d, *J* = 5.7 Hz, 2H), 2.90 (t, *J* = 7.3 Hz, 2H), 2.28 (t, *J* = 7.4 Hz, 2H), 1.63 (dp, *J* = 40.9, 7.2 Hz, 4H), 1.42-1.27 (m, 4H). ^13^C NMR (101 MHz, DMSO-*d_6_*): δ 171.16, 143.11, 139.22, 128.29, 120.98, 117.40, 117.12, 62.90, 36.35, 30.24, 29.28, 28.55, 28.17, 25.06. MS (ESI) m/z for SeSAHA [M + Na] ^+^: 651.1218.

### Synthesis of SeSA-DCA

To a solution of SeSAHA (0.1 g, 0.16 mmol) in anhydrous DMF (1.5 mL) with nitrogen protection, Et_3_N (50 μL) was added at 0 °C. The mixture was stirred at RT for 30 min. DCA (MedChemExpress, HY-10221) (36.5 μL, 0.239 mmol) was added at 0 °C and the mixture was further stirred for 12 h at RT. The mixture was then diluted in ethyl acetate (20 mL) and washed thrice with 5% HCl (20 mL × 3) and saturated NaCl solution (20 mL × 3), respectively. The organic layer was dried over Na_2_SO_4_ and then filtered off. The ethyl acetate was distilled in a vacuum to obtain the crude product. The crude product was purified by column chromatography with silica gel and a gradient of dichloromethane/methanol (200:1) as a mobile phase giving SeSA-DCA as yellow oil (0.097 g, 71% yield). ^1^H NMR (400 MHz, DMSO-*d_6_*): δ 9.94 (s, 1H), 7.66 (t, *J* = 1.9 Hz, 1H), 7.62-7.50 (m, 1H), 7.30 (t, *J* = 7.9 Hz, 1H), 7.06 (dt, *J* = 7.6, 1.3 Hz, 1H), 6.94 (s, 1H), 5.25 (s, 2H), 2.90 (t, *J* = 7.3 Hz, 2H), 2.29 (t, *J* = 7.4 Hz, 2H), 1.61 (dq, *J* = 39.6, 7.3 Hz, 4H), 1.40-1.27 (m, 4H). ^13^C NMR (101 MHz, DMSO-*d_6_*): δ 171.31, 139.56, 135.09, 128.91, 122.85, 119.23, 118.87, 68.59, 64.92, 36.28, 30.19, 29.25, 29.00, 28.50, 28.11, 24.94. MS (ESI) m/z for SeSA-DCA [M + H]^+^: 849.0055.

### Examination of the decomposition of SeSA-DCA in Redox condition

The standard curve of SeSA-DCA was first established by HPLC, HPLC grade acetonitrile/DI water (9:1) was chosen as the eluting solution and the UV wavelengths for the analysis were set at 244 nm. SeSA-DCA was incubated with 10 mM/0.1 mM DTT or 3 mM/0.1 mM H_2_O_2_ in an elute solution for different hours at 37 °C. The solution was then used for HPLC analysis with the established method after filtering by 0.22 μm PTFE membranes. The decomposition ratio was calculated through the peak area and standard curve of SeSA-DCA (n = 3). Column temperature: 35 °C; flow rate: 1.0 mL min^-1^; injection volume: 20 µL.

### Cell culture assay

Human cancer cell lines (PC3, DU145, A549, H1299, MDA-MB-231, HepG2, PANC-1, MIA PaCa-2, HT-29, HCT116, and HeLa) were obtained from the Cell Resource Center, Peking Union Medical College (National Infrastructure of Cell Line Resource, NSTI). These cell lines were cultured according to NSTI culture guidelines and cultured in RPMI 1640 medium (Biological Industries, Israel) supplemented with 10% heat-inactivated fetal bovine serum (FBS; BI; Israel), penicillin/streptomycin (P/S; 100 IU/ml; Gibco, 10378016) in an incubator at 37 °C and 5% CO_2_.

### Cell viability assay

Cell cytotoxicity assays were performed in 96-well plates (NEST) to determine the growth kinetics of SeSA-DCA-treated cells using CCK-8 reagent (Shandong Sparkjade Biotechnology Co., Ltd., CT0001-B). Cells (5000 cells/well) were treated with DMSO or different drugs. Each treatment was performed in five replicates. To assess cell growth, 10 μL of CCK-8 reagent was introduced to every well and allowed to incubate for 4 h. Following that, the absorbance value for each well was measured at 450 nm. Every experiment was duplicated no less than thrice 16.

### Cell cycle arrest analysis

PC3 or DU145 (1 × 10^5^ cells/well) cells were cultured in 6-well plates (NEST Biotechnology) for 48 h before exposure to SeSA-DCA. DCA + SeSAHA, SeSAHA, SAHA (MedChemExpress, HY-10221), DCA, or DMSO (0.5%) for another 48 h duration (Table S1). The cells were then washed twice with PBS. Cell cycle analysis was performed using PI dye per the instructions provided by the manufacturer of the Cell Cycle Staining Kit (Multi Sciences, CCS012). Flow cytometry data were analyzed using ModFit LT 5.0.

### Annexin V-FITC/propidium iodide staining assay

PC3 or DU145 (1 × 10^5^ cells/well) cells were seeded in 6-well plates for 48 h. Cells were treated with SeSA-DCA, DCA + SeSAHA, SeSAHA, SAHA, DCA, or DMSO (0.5%) for 48 h. The FITC-Annexin V/PI apoptosis kit (US EVERBRIGHT, Suzhou, China) was employed to monitor apoptosis in accordance with the manufacturer's guidelines. The flow cytometry assays were conducted using FACS Calibur equipment (BD Biosciences). Flow cytometry data were analyzed using FlowJo X software.

### Colony formation assay

A total of 500 cells were seeded in 6-well plates in triplicate. After the cells adhered to the wall, the medium containing drugs was added, and the liquid was changed every three days. After 10-14 days, crystal violet (Sigma-Aldrich, C0775-25G) staining was performed, and the cells were counted.

### Protein extraction and western blot

Protein extraction and western blot assays were performed as previously described 16. Briefly, cells were lysed in RIPA Buffer (Sperikon Life Science & Biotechnology co., Ltd.) for 30 min at 4 °C. The protein extract was prepared according to the operating guidelines of the BCA Protein Quantification Kit (Jianglai, Shanghai, China). Then, the samples were run on an acrylamide gel in 1 × Tris-glycine electrophoresis buffer. The protein samples were separated on acrylamide gels and then transferred onto a PVDF membrane (Millipore, R1DB89779) using a transfer buffer (20% methanol). The protein samples were detected by immunoblotting with Ki67 (CST, 2586), PCNA (Bioss, bs-2007R), CDK2 (Santa Cruz Biotechnology, sc-6248), CDK4 (Santa Cruz Biotechnology, sc-23896), CDK6 (Abways, CY5835), cyclin D (CST, 55506), cyclin E (Sino Biological Inc., 10902-RP02), Rb (CST, 9309), p-Rb (CST, 8516), Ac-H3 (CST, 8173), tubulin (CST, 5335), cleaved-PARP (c-PARP) (CST, 5625), Bcl-2 (CST, 15071), Bax (CST, 89477), p21 (ZENBIO, 382492), cleaved-caspase3 (c-Casp3) (CST, 9664), ZEB1 (CST, 70512), E-cadherin (CST, 14472), vimentin (CST, 5741), p-PDH (Abcam, ab177461), HDAC1/2/3/5/6/7/8 (Immunoway) , and CDC25A (CST, 3652) antibodies. Using tubulin as a control, band strength was evaluated using ImageJ software.

### TUNEL apoptosis detection

To detect TUNEL cell apoptosis, the TUNEL Cell Apoptosis Detection Kit was utilized following the manufacturer's instructions (Promega, G3250).

### Transwell assay

The cells (5 × 10^4^ cells/chamber) were premixed with drugs or DMSO (0.5%) in 100 µL serum-free RPMI-1640 medium. The chambers were placed in a 24-well plate containing 10% fetal bovine serum and incubated for 24 h. Cells in the chambers were gently wiped off using cotton swabs. After being washed twice with PBS, the chambers were stained with a 0.5% solution of crystal violet for 15 min. The number of cells transferred at the bottom of the filter was observed using a light microscope.

### Wound healing assay

The cells (1 × 10^5^ cells/well) were seeded in a 6-well plate for 24 h. The middle of the attached cells was wiped off using the tip of a sterile 10 µL gun to form a "wound”. After washing with PBS twice, the fresh culture medium containing drugs or DMSO (0.5%) was added to continue treatment for 24 h, and the image of cell migration was observed. The direction of the wounds was recorded using a light microscope.

### *In vivo* tumor growth inhibition (xenograft)

All animal experiments were performed in conformity with guidelines that had been approved by the Experimental Animal Management Committee of Nankai University (2021-SYDWLL-000012). Thirty-five male BALB/c nude mice (four-six weeks old) were raised in the animal room at the School of Life Science, Nankai University. These mice have free access to food and water. PC3 PCa cells (5 × 10^6^) suspension (100 μL) were injected into the right armpits of the mice. Once the mice's tumor volume reached around 50 mm^3^, they were separated into seven groups (n = 5): (a) control group (5% DMSO), (B) DCA (100 mg/kg), (C) SAHA (20 mg/kg), (D) SeSAHA (20 mg/kg), (E) DCA (100 mg/kg) + SeSAHA (20 mg/kg), (F) SeSA-DCA (20 mg/kg), and (G) docetaxel (10 mg/kg) (Glpbio (Montclair, CA, USA)). Tumor size and body weight of tumor-bearing mice in the different groups were measured on the day of injection, two-three days a week. The formula for measuring the size of the tumor was 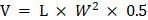, where L and W are the length and width of the tumor, respectively. Fifteen days after administration, the mice were euthanized, and tumor tissues were collected and prepared for HE or IHC staining.

### Immunofluorescence staining

Cells (2 × 10^5^) were seeded in confocal dishes for 24 h. The cells were treated with drugs or DMSO (0.5%) for 48 h. The cells were washed with PBS twice, then fixed for 20 min with 4% paraformaldehyde, and permeated for 5 min with Triton X-100 (0.1%) solution. Blocked with serum albumin of the same genus (5%, w/w) at room temperature (RT), then incubated overnight with the primary antibody at 4 °C, and the fluorescent second antibody was incubated at room temperature for 30 min. DAPI was incubated at 25 °C for 3 min. Imaging was conducted using confocal laser scanning microscopy.

### RNA-sequencing

PC3 cells were seeded at a density of 5 × 10^6^ cells/flask in two 75 cm^2^ flasks. Treatment with drugs for 48 h was performed in one flask, whereas the other was used as a control. Total RNA was isolated using TRIZOL, and the total RNA was extracted from the sample with RNA extraction kit (Accurate Biotechnology (Hunan) Co., Ltd. ChangSha, China). DNA was digested with DNase (GenScript, E00053). Magnetic beads with Oligo (dT) were used to enrich eukaryotic mRNA, and cDNA library construction and sequencing were performed on the Illumina HiSeq Xten platform. After quality inspection, raw data (raw reads) were processed using Trimmomatic. Reads containing ploy-N and low-quality reads were removed to obtain clean reads. The clean reads were then mapped to the reference genome using HISAT2. The FPKM value of each gene was calculated using cufflinks, and the read counts of each gene were obtained by htseq-count. DEGs were identified using DESeq. An adjusted *p*-value < 0.05; |log2foldchange| > 1 was set as the threshold for significantly differential expression. Hierarchical cluster analysis of DEGs was performed to explore the gene expression patterns. KEGG pathway enrichment analysis and PPI and IPA analyses of the DEGs were performed.

### Gene set concentration analysis (GSEA)

GSEA was conducted using GSEA software.

### Quantitative real-time PCR

Total RNA was extracted from the cells using TRIZOL, and cDNA was synthesized by reverse transcription (AE341, TransGen Biotech, China). Real-time PCR was performed using HieffqPCR SYBR ®Green Master Mix (Cat#11201, Yeasen, Shanghai, China). Data were normalized to GAPDH as a loading control. The melting curve and CT value were analyzed using Lightcycler ®96 software. Primer sequences are shown in Table S2.

### Drug toxicity test

Blood samples from each mouse were kept at room temperature for 3 h and centrifuged at 8000 rpm for 15 min to produce serum. The samples were then placed on the blood routine detector for biochemical analysis.

### Molecular docking

The 3D structure of SeSA-DCA was prepared and minimized using Sybyl. The crystal structures of CDC25 (PDBID: 1C25), HDAC1-4 (PDBID: 5ICN, 4LXZ, 4A69, 2VQM) and HDAC6-8 (PDBID: 6VNR, 3C0Z, 1T69) were obtained from the RCSB Protein Data Bank. The 3D structure of HDAC5 (UniProtID: Q9UQL6) and HDAC9 (UniProtID: Q9UKV0) predicted with AlphaFold 17, 18 were downloaded from the UniProt database 19. Molecular dockings were performed using the CB-Dock2 docking server 20.

### *In vivo* histopathological and immunohistochemical studies

The different tissues (liver, lung, spleen, kidney, and tumor) fixed with paraformaldehyde were stained with IHC and HE according to the standard procedure recommended by CST. The diluted HDAC1 (1:200, CST, 4668), p-PDH (1:150, CST, 4551), and CDC25A (1:200, CST, 9145) were added to the tissues and incubated overnight at 4 °C. According to the manufacturer's instructions, a rabbit anti-histochemical kit (ABS957, Absin) was used to label the secondary and tertiary antibodies. Imaging was conducted using a microscope (FV1000, Olympus).

### Statistical analyses

Graphs were generated using GraphPad Prism 8 with default parameters. Data were tested for normality using the Shapiro-Wilk test. Data that were normally distributed were analyzed with 1-way or 2-way ANOVA followed by multiple comparisons performed with LSD test among 3 or more groups, followed by unpaired Student t-tests with the Bonferroni correction between 2 groups. The Kruskal-Wallis 1-way ANOVA on ranks was used for data that were not normally distributed. Statistical significance was set at *p* < 0.05. The figure legend specifies the method and *p*-value for use. All cell-based experiments were performed at least three times in triplicate.

## Results

### Synthesis of SeSA-DCA prodrug

**Figure 1:**
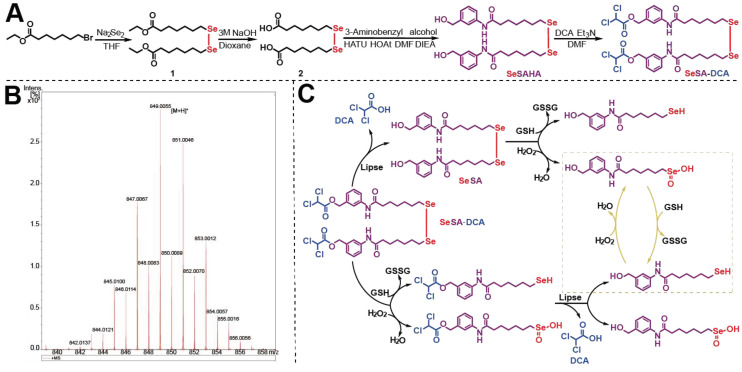
Synthesis and characterization of SeSA-DCA. (A) The synthetic route of SeSA-DCA. (B) MS spectrum of SeSA-DCA. (C) Schematic diagram of redox response fracture of SeSA-DCA.

The synthetic route of SeSA-DCA prodrug is depicted in Figure 1A. The prodrug could be conveniently synthesized in four steps, and the chemical structures of the intermediates (Compounds 1 and 2 and SeSAHA), as well as of the final compounds, were confirmed by ^1^H and ^13^C NMR spectroscopy (Figure S1-4) and mass spectrum (Figure 1B). We also found from Figure S5 that no chemical shift changes occurred around/within the diselenide bond, which was located at 294 ppm in the ^77^Se NMR spectrum. This finding suggests that the ligation of DCA to SeSAHA does not influence the stability of Se-Se linkage.

### Redox response assay of SeSA-DCA

When prodrugs enter the body, due to the different environments between normal cells and cancer cells, they will be oxidized or reduced to produce different compounds in different environments, resulting in drug effects.

To verify the effect of redox conditions on SeSA-DCA, we started by testing SeSAHA and DCA, which are two major ingredients of SeSA-DCA, by HPLC (Figure S6A, B) and created the standard curve of SeSA-DCA, y = 5.67 × 10^7^x + 193812 (R^2^ = 0.9991) by HPLC (Figure S6C). We then investigated the redox-responsive drug decomposition of SeSA-DCA using H_2_O_2_ (a frequently-used analog of ROS) and dithiothreitol (DTT, a frequently-used analog of GSH) as redox agents. We conducted five different simulation experiments: (a) mobile phase control group; (b) 0.1 mM DTT; (c) 10 mM DTT; (d) 0.1 mM H_2_O_2_; (e) 3 mM H_2_O_2_. The GSH (10 mM) and H_2_O_2_ (0.1 mM) concentrations were set to match the biological GSH and ROS levels in the actual cancer microenvironment. DTT (0.1 mM) simulates the normal cellular environment *in vivo*. According to the reduction of the main peak area and the standard curve of SeSA-DCA, the decomposition ratio under different redox conditions was determined.

As can be seen in Figure S6D, SeSA-DCA hardly decomposes in the control group, suggesting that the chromatographic conditions have no effect on SeSA-DCA and will not cause the decomposition and release of drug molecules. In contrast, SeSA-DCA exhibited efficient decomposition after adding DTT and H_2_O_2_, and the oxidation environment played a greater role in promoting its decomposition. When triggered with 3 mM H_2_O_2_, SeSA-DCA showed a distinctly higher decomposition than in other conditions. It can reach 20% decomposition under the action of 3 mM H_2_O_2_ for 48 h, while the decomposition rate is 15% under the condition of 10 mM DTT. In addition, relatively low concentrations of H_2_O_2_ (0.1 mM) and DTT (0.1 mM) decomposition media were tested, and the decomposition rate of SeSA-DCA was not high.

It can be seen from the data in Figure S6D that the decomposition rate of SeSA-DCA was not high in normal cells, while it was high in the tumor environment due to the dual effects of oxidation and reduction. Due to this experiment being an *in vitro* simulation experiment, the concentration of DTT and H_2_O_2_ will decrease during the experiment, while the concentration will remain constant in the *in vivo* environment. Therefore, it can be considered that the decomposition proportion *in vivo* will be slightly higher than in the experimental results.

### SeSA-DCA inhibits the proliferation of PCa cells

**Figure 2:**
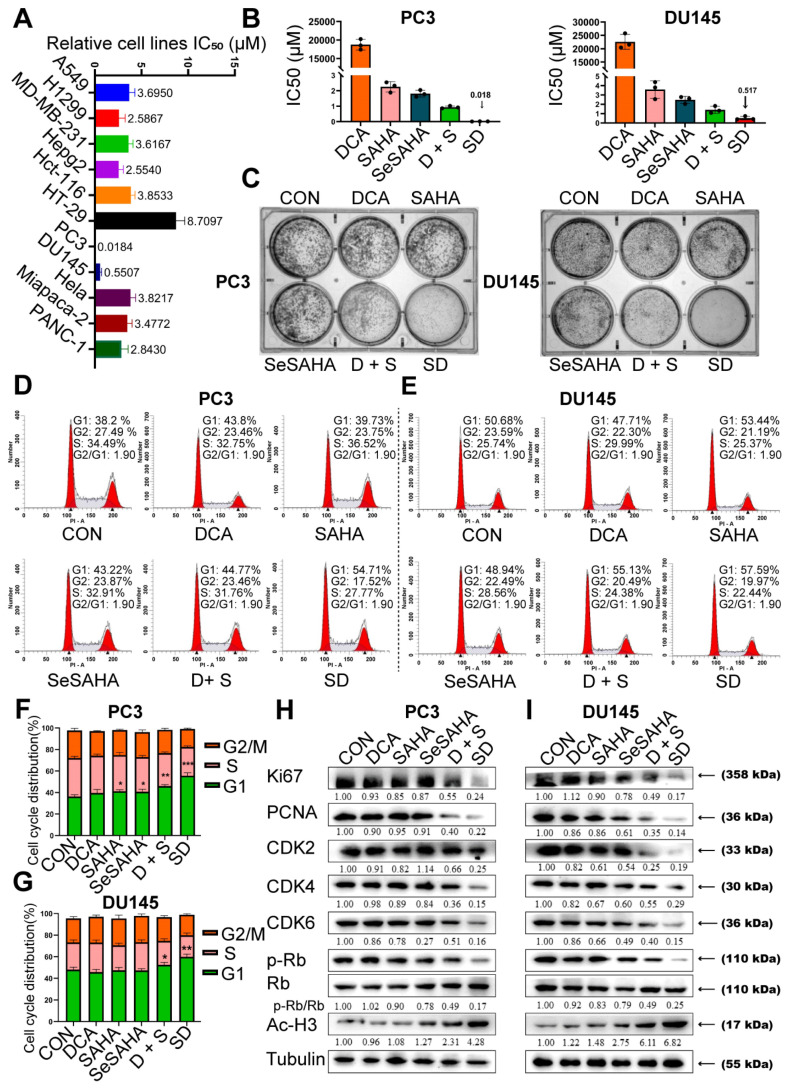
SeSA-DCA inhibits the proliferation of PCa cell lines. (A) SeSA-DCA showed the greatest cytotoxicity in many cancer cell lines. (B) Among all the tested compounds, SeSA-DCA showed the greatest cytotoxicity. (C) Effects of tested compounds on clone formation of PCa cell lines. (D-G) Cell cycle arrest of PCa cell lines by tested compounds. (H-I) The changes in cell cycle and cell proliferation-related proteins in PCa cell lysates were detected by Western blot. Data are shown as the mean ± s.d. *p < 0.05, **p < 0.01, ***p < 0.001. D + S: DCA + SeSAHA; SD: SeSA-DCA.

The inhibition of cell activity of various cell lines by SeSA-DCA, including those for two aggressive PCa cell lines, two non-small cell lung cancer cell lines, two high-grade serous pancreatic cancer cell lines, two colorectal cancer cell lines, one liver cancer cell line, and one cervical cancer cell line, were examined (Figure 2A). It exhibited high anti-cancer activity, with IC_50_ values ranging from 0.018 to 8.7 μM. In particular, SeSA-DCA displayed higher anti-proliferative activity in PC3 cells than in other cancer cell lines, with an approximately 100-fold difference in IC_50_ values (Figure 2A).

In contrast to SeSA-DCA, the reference drugs SeSAHA and DCA had only a minor impact on cell viability at micromolar or millimolar potency. For instance, in PC3 cells, the IC_50_ values of SAHA and SeSAHA were higher than 1.8 μM, and DCA was higher than 1.8 × 10^4^ μM. The physical combination of DCA and SeSAHA treatment showed a slightly lower IC_50_ value than the SeSAHA group (approximately 1.0 μM). Surprisingly, the IC_50_ value of SeSA-DCA (0.018 μM) was almost two orders of magnitude lower than that of all reference drugs, including the physical combination group. The same trend was observed in DU145 cells (Figure 2B). Therefore, these outcomes propose that the SeSA-DCA hybrid approach demonstrates a significant enhancement in comparison to the reference compounds. We also compared anti-tumor activity on PCa cells viability between SeSA-DCA and docetaxel, a clinical first-line drug for PCa. The results demonstrated that the IC_50_ values of the PC3 cells were nearly identical.

To assess the capacity of individual cells to proliferate, a colony formation test was conducted. In all PCa cell lines, SeSA-DCA inhibited long-term proliferation to a greater extent than each inhibitor alone or in combination at the same dose (Figure 2C). In addition, the proliferation-related proteins, Ki67 and PCNA, were evidently downregulated by SeSA-DCA treatment.

### SeSA-DCA induced G1 arrest in PCa cells

Cell cycle arrest inhibits cell proliferation and is accompanied by cell death. Given the evidence that both DCA and SAHA can modulate the cell cycle phase distribution in different cancer cells, we subsequently evaluated the impact of SeSA-DCA on cell cycle regulation in PCa cells. PCa cells were exposed to SeSA-DCA at concentrations corresponding to the IC_50_ values for 48 h. The cell cycle analysis revealed that, in all cell lines, SeSA-DCA elevated the proportion of cells in the G1 phase and reduced the percentage of cells in the S phase when compared to DMSO and reference compounds (SAHA, SeSAHA, DCA alone, and a combination therapy) (Figure 2D-G). In line with the cell cycle outcomes, we discovered that SeSA-DCA caused a considerable decline in CDK4, CDK6, CDK2, cyclin D, cyclin E, and Rb phosphorylation following *in vitro* exposure (Figure 2H, I), which might account for G1-phase arrest. On the other hand, the equivalent dosages of SeSAHA and DCA did not cause G1 to S phase arrest or modify the expression of proteins associated with the cell cycle. Even when compared with the equimolar mixture of SeSAHA and DCA, the inhibition of the expression of these key proteins by SeSA-DCA was more than two-fold (Figure 2H, I).

### SeSA-DCA promotes apoptosis of PCa cells

**Figure 3:**
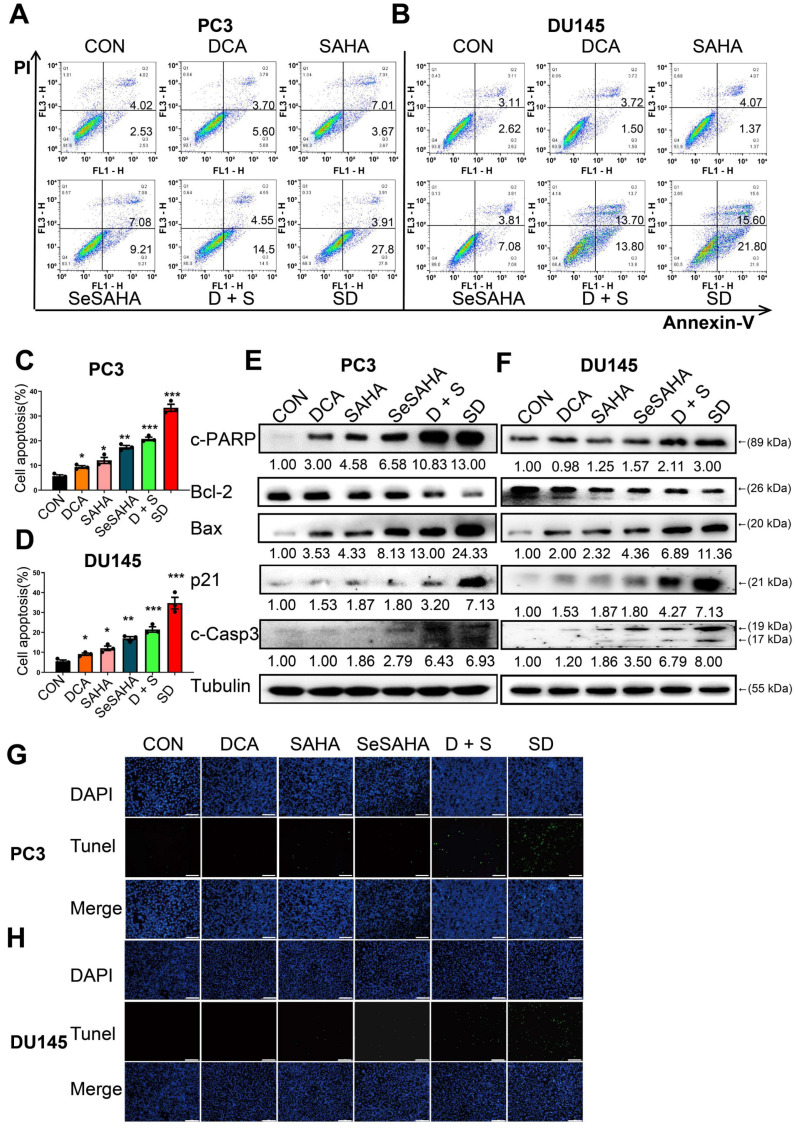
SeSA-DCA induces cell apoptosis in PCa cell lines. (A-D) The ability of the tested compounds to induce apoptosis of PCa cell lines was detected by flow cytometry. (E, F) Western blotting of apoptosis-related proteins in PCa cell lysates by tested compounds. (G, H) The ability of the tested compounds to promote apoptosis of PCa cell lines was detected by TUNEL, the scale is 200 μm. Data are shown as the mean ± s.d. *p < 0.05, **p < 0.01, ***p < 0.001. D + S: DCA + SeSAHA; SD: SeSA-DCA.

We performed flow cytometry analysis to investigate if SeSA-DCA induces apoptosis and contributes to its anti-cancer effects in PCa cells. PC3 and DU145 cells were treated with SeSA-DCA, reference drugs alone or in combination, and vehicle (*dimethylsulfoxide*; DMSO) for 48 h, followed by Annexin V/propidium iodide (PI) staining. The percentage of apoptotic cells responding to SeSA-DCA was notably greater than that caused by the positive controls at equivalent concentrations (Figure 3A-D).

To provide additional evidence for SeSA-DCA inducing apoptosis, we analyzed the levels of apoptotic proteins in these cancer cell lines. SeSA-DCA led to a significant induction of Bax, c-PARP, and c-Caspase-3 while decreasing the level of Bcl-2 and, in particular, the ratio of Bcl-2/Bax (Figure 3E, F). In addition, SeSA-DCA showed a more substantial effect than reference drugs alone or in combination. The observed changes were mainly linked to the maintenance of HDAC activity in response to treatment with SeSA-DCA. Moreover, p21 functions as a cyclin-dependent kinase inhibitor that controls apoptosis and is targeted by HDAC. In both cell lines, the groups treated with SeSA-DCA exhibited an elevation in the expression of p21. The above information suggested that PCa cell lines undergo apoptosis as a result of SeSA-DCA treatment.

The TUNEL assay also confirmed that SeSA-DCA induced apoptosis of PCa cell lines, and the results were consistent with the flow cytometry analysis (Figure 3G, H). The apoptosis of cancer cells induced by SeSA-DCA was the most obvious, whereas that of the other groups was much lower.

### SeSA-DCA inhibits metastasis and invasion of PCa cells *in vitro*

**Figure 4:**
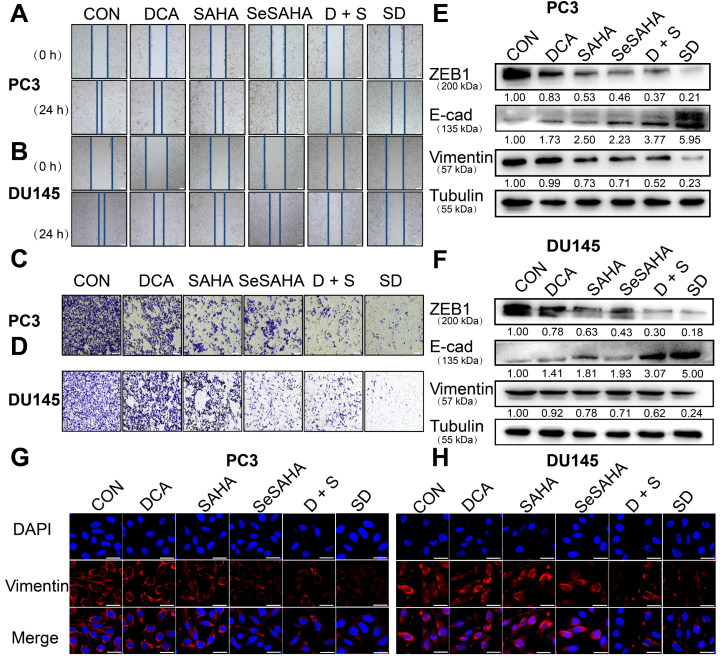
SeSA-DCA inhibits metastasis and invasion of PCa cell lines. (A-B) Scratch test showed that the tested compounds could inhibit the migration of PCa cell lines, the scale is 200 μm. (C-D) Transwell assay showed that the tested compounds inhibited the migration and invasion of PCa cell lines, the scale is 200 μm. (E-F) Western blotting of tested compounds on invasion and metastasis-related proteins in the lysate of PCa cell lines. (G-H) Cellular immunofluorescence assay showed the effect of tested compounds on the expression of Vimentin protein in PCa cell lines, the scale is 20 μm. D + S: DCA + SeSAHA; SD: SeSA-DCA.

The wound healing scratch test and Transwell test were used to determine whether SeSA-DCA can inhibit cancer cell invasion and metastasis. Compared with the other control groups, the migration ability of PC3 and DU145 cells treated with SeSA-DCA was significantly hindered after 24 h, while the other control groups almost completely migrated to the wound area (Figure 4A, B). The Transwell test was used to evaluate the anti-metastatic properties of SeSA-DCA. Compared with other control cells, the number of cells that invaded through the capsule in the SeSA-DCA-treated group was very low (Figure 4C, D). Additionally, Western blotting was performed to examine the expression of proteins associated with epithelial-mesenchymal transition (EMT). The expression of ZEB1 and vimentin in SeSA-DCA cells was significantly decreased, and E-cadherin was also upregulated (Figure 4E, F). Similarly, we evaluated the expression of vimentin proteins by cellular immunofluorescence staining, and the results were consistent with the above (Figure 4G, H). The results indicated that both SeSA-DCA and an equimolar mixture of SeSAHA and DCA effectively inhibited the EMT process of PCa, and SeSA-DCA exhibited a superior synergistic effect.

### SeSA-DCA significantly inhibits the growth of the transplanted tumor with low toxicity

**Figure 5:**
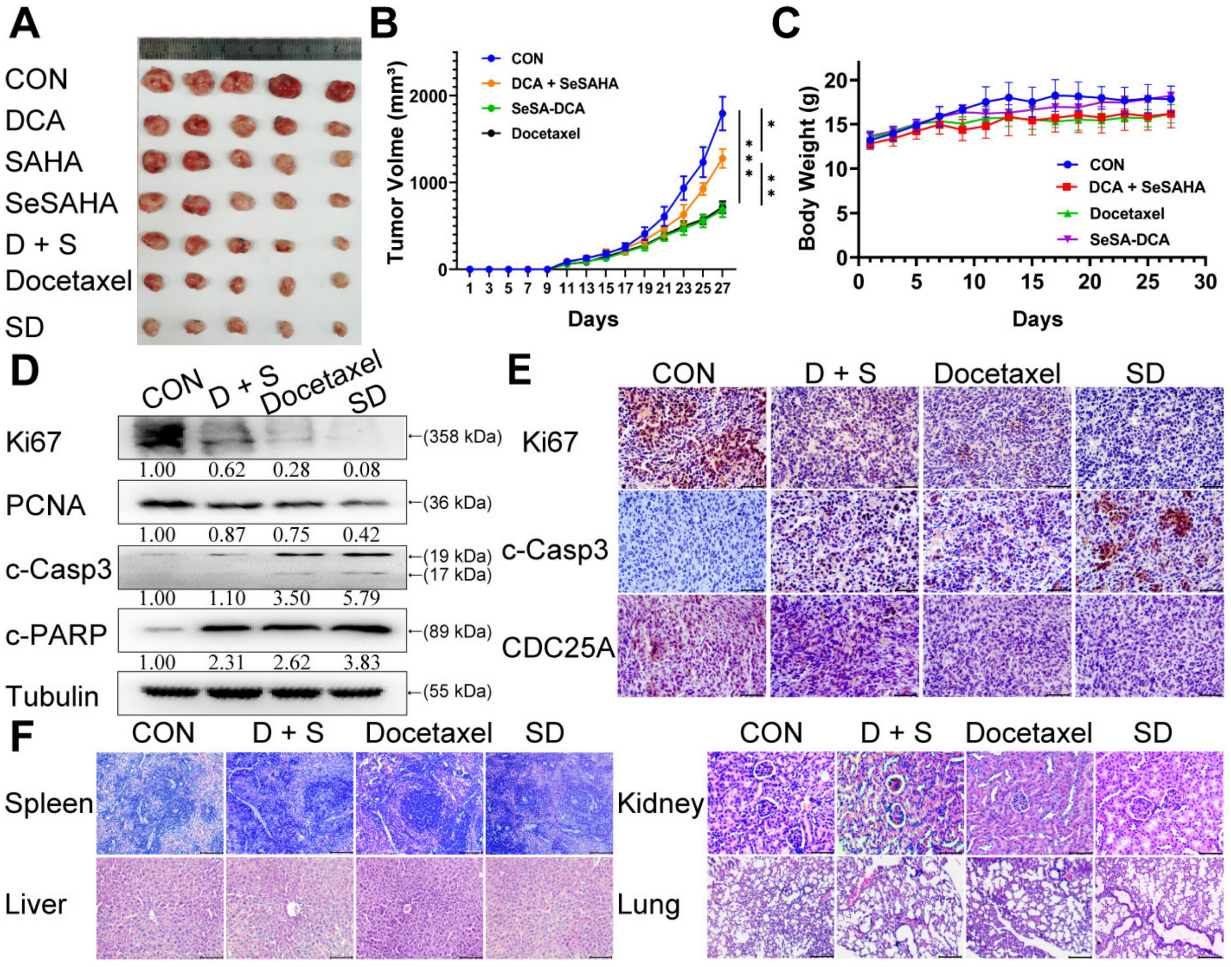
SeSA-DCA inhibits the growth of transplanted tumor and its mechanism. (A) Photographs of PC3 xenograft tumors. Mice were sacrificed 15 days after tumor implantation. (B) Tumor growth curve of PC3 tumor-bearing mice in response to different treatments. (C) Effects of different treatments on body weight growth curve of PC3 tumor-bearing mice. (D) Western blotting confirmed the changes in the proliferation and apoptosis proteins in PC3 transplanted tumor. (E) Immunohistochemical study on the changes of Ki67, c-Casp3, and CDC25A protein in PC3 xenografts, the scale is 100 μm. (F) HE staining of spleen (The scale is 200 μm), liver (The scale is 200 μm), kidney (The scale is 50 μm), and lung (The scale is 200 μm). Data are shown as the mean ± s.d. *p < 0.05, **p < 0.01, ***p < 0.001. D + S: DCA + SeSAHA; SD: SeSA-DCA.

To assess the efficacy of SeSA-DCA against tumors in a living organism, we created a mouse model with xenograft PCa and observed its effects. Apart from DCA, SAHA, SeSAHA, and SeSA-DCA groups and a control group, we established two other groups: one with a physical mixture of SeSAHA and DCA and a positive group with docetaxel via intraperitoneal injection. After treatment with SeSA-DCA for 16 days, tumor proliferation in nude mice was effectively decreased, and compared to the other treatments, SeSA-DCA displayed notably superior inhibition. The tumor size in the SeSA-DCA-treated group was even smaller than that in the docetaxel group (Figure 5A, B, Figure S7A). No significant alterations were observed in the body weight of the seven groups of mice (Figure 5C Figure S7B). Additionally, the hemogram findings from the mouse trials indicate that SeSA-DCA had low toxicity towards the circulatory system and normal tissues (Table 1), as suggested by the barely noticeable impact on primary organs such as the liver, spleen, lungs, and kidneys (Figure 5F, Figure S7C). The tumor tissues were then analyzed by Western blotting to detect the different expression levels of proliferation (Ki67 and PCNA), apoptosis (c-Casp3, c-PARP) (Figure 5D), metastasis (vimentin), related substrate (p-PDH, HDAC1) (Figure S7E), and cell cycle-related proteins (CDC25A, CDK4, CDK6, and Rb) (Figure S7E) in each group. These results are consistent with those at the cell level. Immunohistochemistry showed that Ki67, CDC25A (Figure 5E), p-PDH, and HDAC1 (Figure S7D) were significantly downregulated and c-Casp3 (Figure 5E) was significantly upregulated in the SeSA-DCA-treated group compared to the other groups. Collectively, our data illustrated that SeSA-DCA displays significant anti-tumor effects against human prostate tumors *in vivo*, surpassing even the efficacy of docetaxel.

### RNA-seq results of PC3 cells treated with SeSA-DCA

**Figure 6:**
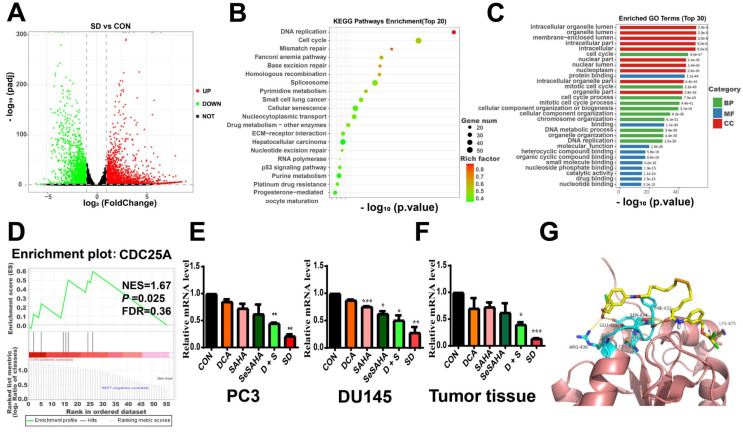
Selection and verification of potential targets for SeSA-DCA. (A) Volcanic diagram of genetic changes of SAHA compared with CON in PC3 cell line. (B) KEGG enrichment analysis of top 20 showing the results of RNA-seq sequencing. (C) GO enrichment analysis TOP 30 terms based on RNA-seq data showing in PC3 cell line. (D) GSEA analysis for enrichment of CDC25A in PC3 cell line. (E) RT-PCR verification of CDC25A expression in PCa cell lines treated with tested compounds. (F) RT-PCR verification of CDC25A expression in tumor tissue treated with tested compounds. (G) Binding mode of SeSA-DCA to CDC25A. Hydrogen bonds between SeSA-DCA and CDC25A are represented by dashed lines, yellow is SeSA-DCA, blue is the residue of the hydrogen bond between CDC25 and SeSA-DCA, and yellowish brown is CDC25A. Data are shown as the mean ± s.d. *p < 0.05, **p < 0.01, ***p < 0.001. D + S: DCA + SeSAHA; SD: SeSA-DCA.

To further explore the underlying molecular mechanisms of the anti-cancer effects of SeSA-DCA, we performed RNA sequencing studies on PC3 cells treated with DMSO, DCA, SAHA, or SeSA-DCA for 48 h. In general, the TIN score (RSeQC v3.0.1) indicated high sample quality, and the biological replicates were closely grouped based on transcriptional changes observed in principal component analysis (PCA; R v3.5). This result suggests that the transcriptomes of SeSA-DCA-treated cells were distinct from those of all other treatment groups. Compared with DMSO, DCA induced minimal changes in gene expression (Figure S8A), whereas SAHA treatment resulted in many changes in gene expression, with 2,024 upregulated and 1573 downregulated transcripts (Figure S8B). SeSA-DCA treatment resulted in even more substantial changes in PC3 cell transcription, with 2639 upregulated and 2180 downregulated genes (adjusted *p*-value < 0.05; |log_2_foldchange| > 1) (Figure 6A).

Using the RNA-seq data, we conducted analyses of Gene Ontology (GO) and Kyoto Encyclopedia of Genes and Genomes (KEGG) pathways for all genes with differential expression. In the biological processes domain, a large proportion of genes were associated with the cell cycle, mitotic cell cycle, and cell cycle processes (Figure 6B). Functional enrichment analysis by mapping differentially expressed genes (DEGs) to the KEGG database indicated that SeSA-DCA treatment could induce various anti-cancer signaling activities. The top three enriched pathways ranked by significance were DNA replication, cell cycle, and mismatch repair (Figure 6C). The GO and KEGG pathway analysis results revealed that cell cycle processes might be an important aspect of SeSA-DCA effects. We found that many key cell-cycle genes were downregulated in PC3 cells after SeSA-DCA treatment; thus, a network statistical analysis was conducted on the PPI of genes associated with cell-cycle processes (Figure S8C, D). A gene set enrichment analysis was carried out using the RNA-seq dataset. We found that the CDC25A signal was significantly enriched in PC3 cells treated with SeSA-DCA (NES = 1.67; *p* = 0.025) (Figure 6D). We confirmed the downregulation of CDC25A by verification of RT-PCR experiments *in vivo* and *in vitro* (Figure 6E, F).

Moreover, the molecular docking studies demonstrated the anticipated binding patterns and intricate interactions between SeSA-DCA and CDC25A (Figure 6G). Six residues (AGR-436, CYS-430, GLU-431, SER-434, PHE-432, and LYS-475) play important roles in the binding of CDC25A and SeSA-DCA. This close binding of SeSA-DCA to CDC25A may explain the inhibitory activity of SeSA-DCA on CDC25A. This confirmed that CDC25A is an important target of SeSA-DCA.

### SeSA-DCA Regulating the Expression of CDC25A in Various Ways to Inhibit Tumor Proliferation

**Figure 7:**
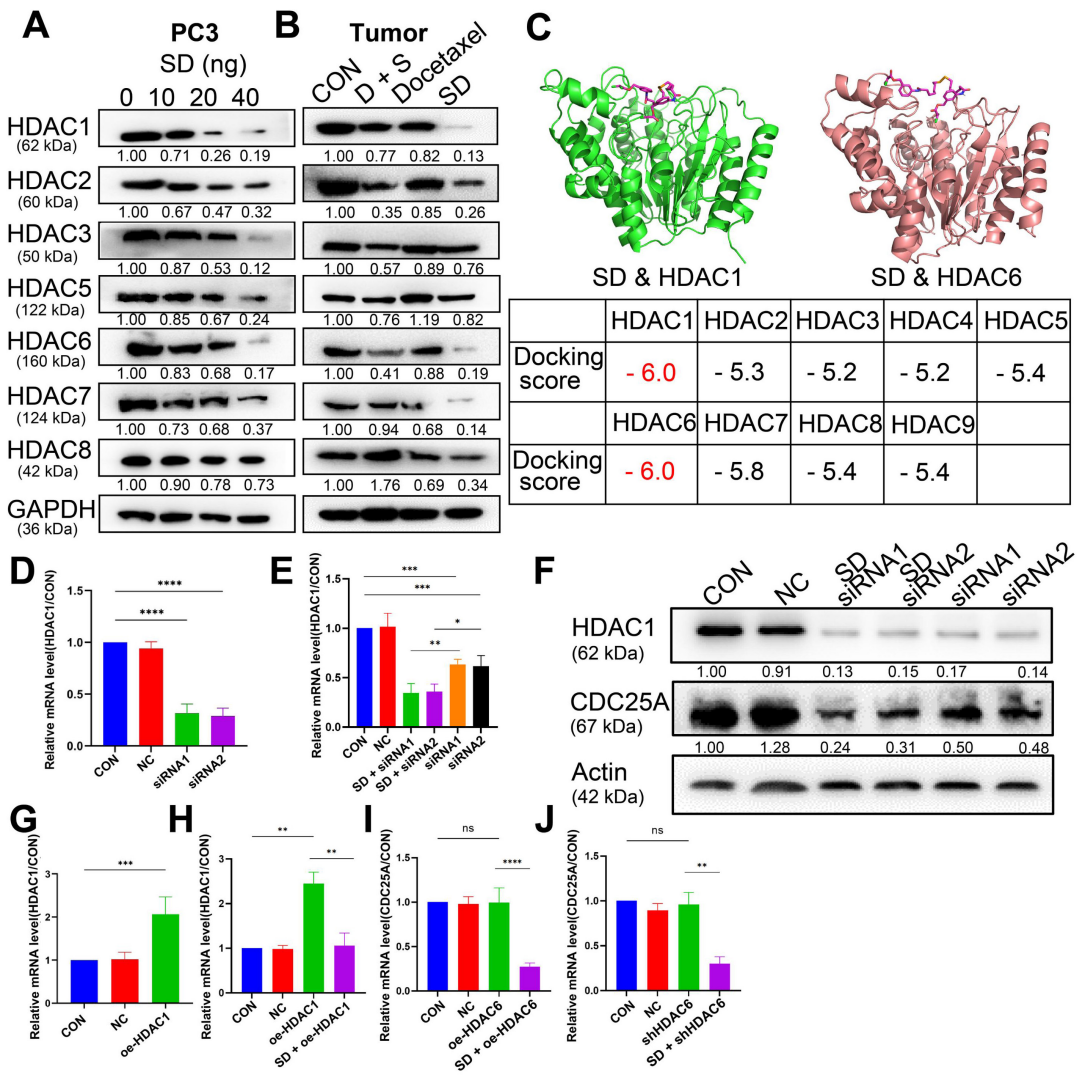
SeSA-DCA regulates CDC25A in many ways to inhibit tumor growth. (A, B) SeSA-DCA effectively reduces the expression of HDACs in PCa cell lines and tumor tissues. (C) Molecular docking results of SeSA-DCA and HDACs. (D) RT-PCR results showed that HDAC1 was knocked down by siRNA in PCa cell lines. (E) RT-PCR results showed that CDC25A transcription was affected by siRNA-HDAC1 and SeSA-DCA treatment in PCa cell lines. (F) Western blotting showed that CDC25A transcription was affected by siRNA-HDAC1 and SeSA-DCA treatment in PCa cell lines. (G) RT-PCR results showed that HDAC1 was overexpressed in PCa cell lines. (H) RT-PCR results showed that CDC25A transcription was affected by oe-HDAC1 and SeSA-DCA treatment in PCa cell lines. (I) RT-PCR results showed that CDC25A transcription was affected by shRNA-HDAC6 and SeSA-DCA treatment in PCa cell lines. (J) RT-PCR results showed that CDC25A transcription was affected by oe-HDAC6 and SeSA-DCA treatment in PCa cell lines. Data are shown as the mean ± s.d. *p < 0.05, **p < 0.01, ***p < 0.001. D + S: DCA + SeSAHA; SD: SeSA-DCA.

We assumed that the anti-cancer activity of SeSA-DCA involves SAHA and DCA. Therefore, we tested the variation of SAHA and DCA substrate. According to the test results, SeSA-DCA inhibited the expression of HDACs in both class I (1, 2, 3, 8) and class II (4, 5, 6, 7, 9, 10) in PCa cell lines (Figure 7A, B). After 20 nM SeSA-DCA treatment, class I HDAC1 and class II HDAC6 were most significantly inhibited (Figure 7A, B). Similarly, in the molecular docking results of SeSA-DCA and HDACs, the binding energy of HDAC1 and HDAC6 is the lowest. It shows that SeSA-DCA inhibits HDAC1 and HDAC6 most obviously (Figure 7C, Figure S9). Besides, SeSA-DCA also greatly reduced the expression of p-PDH, a substrate of DCA. On the whole, SeSA-DCA demonstrated the most effective inhibition to the substrate of all groups (Figure S7A, B).

Previous research showed that HDAC1 has an impact on the transcriptional level of CDC25A 21. We found that SeSA-DCA inhibited the expression of CDC25A in both transcriptional level and translational level (Figure 6E, F, Figure 7SA, B). We conducted experiments to further investigate its molecular mechanism. The RT-PCR experiment suggested that CDC25A was remarkably down-regulated after the PC3 cell line being processed with si-HDAC1, and even more so when the cell line had been treated by SeSA-DCA (Figure 7D, E). The Western blot test indicated consistent results (Figure 7F). In addition, CDC25A was significantly up-regulated with the overexpression of HDAC1 but down-regulated after the SeSA-DCA treatment in cell lines (Figure 7G, H). We also investigated the impact of HDAC6 on CDC25A and found that CDC25A was free of any influence from either the knockout or the overexpression of HDAC6 (Figure 7I, J). In summary, SeSA-DCA can inhibit the transcription and translation of CDC25A by containing the expression of HDAC1. Combining the test results of molecular docking between SeSA-DCA and CDC25A (Figure 6G), we have concluded that SeSA-DCA can inhibit the expression of CDC25A not only by direct interaction with it but also by regulation of HDAC1, therefore causing the cell cycle arrest of prostate cancer cells and impeding their proliferation.

## Discussion

The approach to treating cancer has evolved from traditional chemotherapy to a more targeted strategy based on underlying mechanisms. HDACs play a role in governing gene expression, and their abnormal recruitment and overproduction in cancer cell lines make them critical targets for anti-cancer interventions. HDAC inhibitors have a greater impact on tumor cells than normal cells, as they selectively modify gene expression, leading to the activation of multiple anti-tumor pathways. Vorinostat, also known as suberoylanilide hydroxamic acid (SAHA), is a potent inhibitor of class I and II HDACs and has been implemented in various clinical trials for cancer treatment, including solid tumors, hematological malignancies, and other advanced cancers. However, the brief half-life of SAHA in biological systems may be attributed to metabolic instability and pharmacokinetic difficulties, such as glucuronide and sulfate conjugates. Hence, numerous novel HDAC inhibitors that are non-hydroxamic have been documented in the literature 22-24. Among these, FK228 is a robust HDAC inhibitor approved by the FDA, with a disulfide bond in its molecule that can be reduced in a cellular environment, liberating the active species in the form of a free thiol analog 25. Se and S are known to belong to the same main group in the periodic table, and Se is more polarizable than S; hence, we hypothesized that in the tumor microenvironment, the selenium dimer could be reduced, and free SeH could be released more quickly than the disulfide compound. Therefore, we synthesized SeSAHA and explored its application in cancer therapy. Preliminary results showed that SeSAHA had a greater inhibitory effect (> 10-fold) than SAHA.

Although preclinical studies have shown encouraging progress, the majority of HDAC inhibitors that are currently known and used as standalone treatments have been unsuccessful in demonstrating any clinical advantages for solid tumors 26. It is imperative to promptly create a combination of HDAC inhibitors, along with chemotherapy or other targeted therapies, to improve clinical effectiveness in the treatment of solid tumors. Therefore, we designed and synthesized a novel prodrug, SeSA-DCA, which conjugated the pyruvate dehydrogenase kinase inhibitor DCA to SeSAHA through a simple esterification reaction. When we treated various cancer cells with SeSA-DCA, it exhibited significant toxic effects on different cancer cells, especially PCa cell lines (Figure 2A). In the PC3 cell line, the IC_50_ value was 18 nM, at least 100 times lower than that of the reference drugs. In animals, SeSA-DCA was found to be more effective at inhibiting tumors than docetaxel, a first-line clinical drug for PCa (Figure 5A, B). Its low toxicity is the cause of the observed tolerability during *in vivo* tests and the absence of any notable decrease in body weight. To our knowledge, such selenium-containing prodrugs have not yet been reported in the literature.

To understand the mechanism by which SeSA-DCA inhibits the proliferation of PCa cell lines, RNA-seq and pathway analyses of differentially expressed genes disturbed by SeSA-DCA treatment were performed. The findings demonstrated that pathways linked to the cell cycle, including DNA replication, were notably enriched. Gene cluster analysis revealed that SeSA-DCA suppressed the transcription of a significant proportion of cell cycle-related genes in PCa cell lines. Consequently, this might lead to the stalling of cells in the G1 phase, which can prevent cell growth and ultimately result in cell death. Previous studies have shown that DCA might induce altered cell cycle distribution or cell cycle arrest at the G1/S checkpoint in many cancer cells. These include endometrial cancer and non-small cell lung cancer (NSCLC) 27. The HDAC inhibitor SAHA also induces G1 cell cycle arrest and apoptosis in multidrug-resistant sarcoma, hepatocellular carcinoma, cholangiocarcinoma, and lymphoma cells, perhaps through alterations of cyclins and associated cycle-dependent kinases 28-30. In our study, SeSA-DCA induced greater G1 cell cycle arrest and apoptosis in PCa cell lines than DCA, SAHA, SeSAHA, or a mixture of SeSAHA and DCA. This might be because SeSA-DCA largely increased the expression level of p21 protein, a target of HDAC, which has a crucial function in controlling the G1/S phase transition of the cell cycle. In addition, the CDC25A-CDK4/6 (CDK2)-cyclin D1(cyclin E)-Rb pathway is involved.

CDC25A is a phosphatase that plays a critical role in the transition from the G1 phase to the S phase of the cell cycle, and it can target both tyrosine and serine/threonine residues. It is connected with the dephosphorylation and stimulation of cell cycle kinases, such as cyclin E-CDK2 and cyclin D-dependent kinases (CDK4 and CDK6) 31. The Myc oncogene also upregulates CDC25A. It can increase glycolysis activity by regulating the key enzymes of glycolysis and providing more energy to promote the rapid division and proliferation of tumor cells 32. It has been reported that SAHA down-regulates the CDC25A mRNA level in MCF-7 cells 33. In this report, we proposed a novel mechanism by which our new prodrug, SeSA-DCA further reduced the expression level of CDC25A (Figure 6A-F; Figure S7E). In the computer simulation of the binding model of CDC25A and SeSA-DCA, six residues (AGR-436, CYS-430, GLU-431, SER-434, PHE-432, and LYS-475) of CDC25A formed six hydrogen bonds with SeSA-DCA, which firmly bound CDC25A and SeSA-DCA. Combined with the docking results of the computer simulations, we assumed that this might be one of the reasons why the prodrug works. When the prodrug is taken up by the tumor cells, it can bind CDC25A in the entity and be engaged in the normal transportation process of the cell.

The lack of CDK4 and CDK6 phosphorylation leads to a decrease in p-Rb, leading to G1 cell cycle arrest and apoptosis. The non-phosphorylated Rb binds to the transcriptional regulatory factor E2F, which results in transcriptional inhibition of the target gene by E2F and cannot activate the genes needed for the S phase. Cells experience G1 cell cycle arrest and initiate apoptosis 34. Some studies have shown that the interaction between HDAC1 and Rb regulates the inhibition of related genes to regulate cell proliferation and differentiation. In an earlier study, it was discovered that E2F activation occurs through the cyclin E-CDK2 pathway, and this factor counteracts the inhibition of CDK4/6, which results in the progression of the cell cycle from the G1 to S phases 35. However, in our study, both the cyclinE-CDK2 axis and CDK4/6 were inhibited by SeSA-DCA. This would further induce G1 cell cycle arrest and apoptosis in PCa cell lines. There is also evidence that CDK2 and cyclin E can be inhibited by CDC25A, affecting Rb phosphorylation and progression from G1 to S phase 36.

Apoptosis is composed of two primary pathways: the first pathway is activated by external factors through death receptors, and the second pathway is activated internally through mitochondria 37. SeSA-DCA treatment significantly upregulated the expression of Bax and downregulated that of Bcl-2 in the two PCa cell lines (Figure 3E, F). The TUNEL assay enabled further examination of DNA fragmentation. As expected, SeSA-DCA significantly increased DNA fragmentation within the nuclei of both cell lines compared to the control group (Figure 3G, H). Furthermore, genes associated with DNA fragmentation, including caspase-3 and PARP, decreased their expression. Caspase-3 is cut through SeSA-DCA processing, causing PARP involved in DNA repair to be cut 38, 39 (Figure 3E, F). These findings suggest that SeSA-DCA induces apoptosis in PCa cell lines, mainly by increasing DNA fragmentation through the intrinsic apoptotic pathway.

EMT is a crucial factor in tumor invasion and metastasis 40, 41. During EMT, epithelial cells lose their cellular polarity and intercellular adhesion; eventually, they become mesenchymal cells and acquire migratory and invasive properties 42. SeSA-DCA significantly inhibited the migration of PCa cell lines, as shown by the wound healing scratch and Transwell assay (Figure 4A-D). SeSA-DCA also significantly inhibited the expression of ZEB1, vimentin, and upregulated E-cadherin (Figure 4E, F). Downregulation of vimentin was confirmed by cellular immunofluorescence (Figure 4G, H). The study outcomes indicate that SeSA-DCA can effectively impede the invasion and metastasis of PCa cell lines *in vitro*.

## Conclusion

In summary, our *in vivo* and *in vitro* results showed that SeSA-DCA is a highly effective anti-tumor compound for PCa. It can effectively induce cell cycle arrest and growth suppression and inhibit the migration and metastasis of PCa cell lines compared with monotherapy. In addition, SeSA-DCA inhibited tumor growth in tumor-bearing mice better than docetaxel. Our research shows that SeSA-DCA is a novel prodrug and a promising therapeutic strategy for PCa treatment.

## Supplementary Material

Supplementary figures and tables.

## Figures and Tables

**Figure 8:**
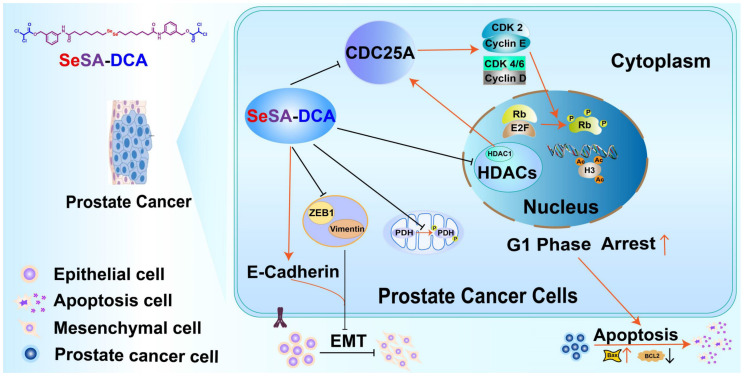
General map of potential anticancer molecular mechanism of SeSA-DCA.

**Table 1:** Effect of drug therapy on blood biochemistry in nude mice.

Parameters	DMSO	DCA (100 mg/kg)	SAHA (20 mg/kg)	SeSAHA (20 mg/kg)	DCA + SeSAHA (100 mg/kg + 20 mg)	Docetaxel (10 mg/kg)	SeSA-DCA (20 mg/kg)
ALT	45.3 ± 5.6	37.5 ± 4.5	53.5 ± 5.5	49.3 ± 6.7	57.1 ± 4.8	46.4 ± 5.7	43.7 ± 4.8
AST	229.8 ± 34.8	223.4 ± 35	285.3 ± 40.6	235.5 ± 40.3	274.4 ± 35.2	251.8 ± 48.3	174.6 ± 27.4
ALP	32.2 ± 8.3	36.2 ± 3.6	34.3 ± 3.3	36.5 ± 4.3	42.6 ± 2.6	34.2 ± 8.2	36.8 ± 5.1
TP	51.9 ± 2.2	51.5 ± 0.45	51.7 ± 2.5	53.7 ± 0.3	52.6 ± 0.5	52.8 ± 4.1	53.5 ± 4.2
GLOB	36.8 ± 3.5	37.1 ± 0.22	35.9 ± 3.6	38.4 ± 1.1	35.8 ± 2.2	38.3 ± 3.1	36.3 ± 3.8
BUN	7.1 ± 2.1	10.5 ± 0.88	7.9 ± 1.5	6.7 ± 0.7	11.5 ± 2.7	9.8 ± 0.8	8.4 ± 0.3
ALB	15.2 ± 1.5	13.45 ± 0.18	15.8 ± 0.6	15.4 ± 2.1	15.5 ± 0.94	14.6 ± 0.6	16.2 ± 1.8
TBIL	0.8 ± 0.2	0.49 ± 0.06	0.8 ± 0.17	0.6 ± 0.07	0.59 ± 0.06	0.7 ± 0.07	0.4 ± 0.03
A/G	0.42 ± 0.03	0.36 ± 0.02	0.4 ± 0.05	0.4 ± 0.07	0.41 ± 0.03	0.38 ± 0.04	0.5 ± 0.05

## Abbreviations

CDK: cyclin-dependent kinases
EMT: epithelial to mesenchymal transition
GSEA: gene set enrichment analysis
HE: hematoxylin and eosin
HDAC4, 6: histone deacetylase 4, 6
HDACi: HDAC inhibitor
IHC: immunohistochemistry
PCa: prostate cancer
RT-PCR: quantitative real-time polymerase chain reaction
SAHA: suberoylanilide hydroxamic acid (vorinostat)
