# Supplementary material for: A novel selenium analog of HDACi-based twin drug induces apoptosis and cell cycle arrest via CDC25A to improve prostate cancer therapy
Source: Theranostics. 2024 Jun 3;14(9):3565–82. doi: 10.7150/thno.92119 (PMC11209715; doi:10.7150/thno.92119)
Supplement: Supplementary file 1 — Supplementary figures and tables. [file thnov14p3565s1.pdf]

A novel selenium analog of HDACi-based twin drug induces apoptosis and cell cycle arrest via CDC25A to improve prostate cancer therapy

Zhiyong Shi[1#], Miaomiao Liu[1#], Xiaowen Zhang[1], Jingyang Wang[1], Junwei Zhang[1], Zeyan Peng[1], Li Meng[1], Ruijing Wang[1], Lihong Guo[2], Qiang Zhang[3], Jing Li[1], Liang Yang[1], Jie Liu[1], Yang Xu[1], Jie Yan[1], Jianlin Cui[1], Shan Ren[4], Yang Gao[1,5*], Yanming Wang[1*], Zhi Qi[1,2,4,5*]

Supplementary Figures and Legends

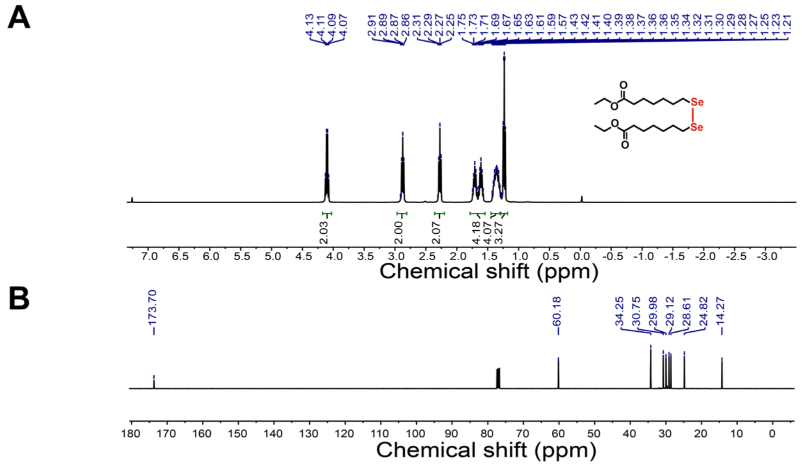

Figure S1. Structure confirmation of Compound 1 in $CDCl_3$. (A): ^1H NMR spectrum of Compound 1 (400 MHz, $CDCl_3$). (B): ^{13}C NMR spectrum of Compound 1 (101 MHz, $CDCl_3$).

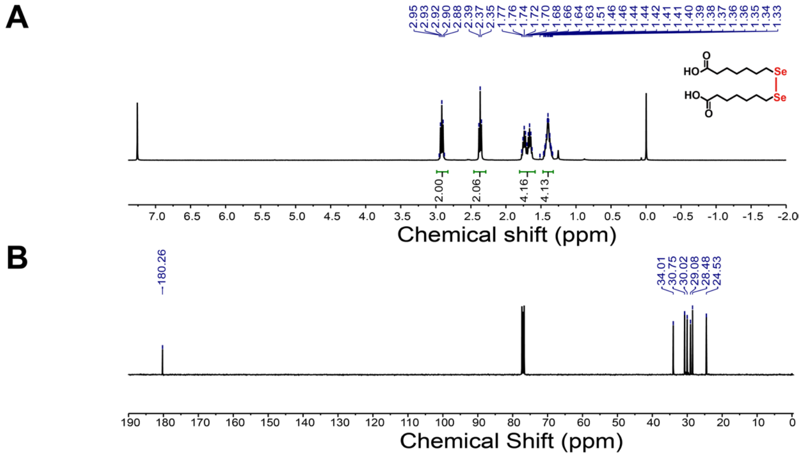

Figure S2. Structure confirmation of Compound 2 in $CDCl_3$. (A): ^1H NMR spectrum of Compound 2 (400 MHz, $CDCl_3$). (B): ^{13}C NMR spectrum of Compound 2 (101 MHz, $CDCl_3$).

A
B
C

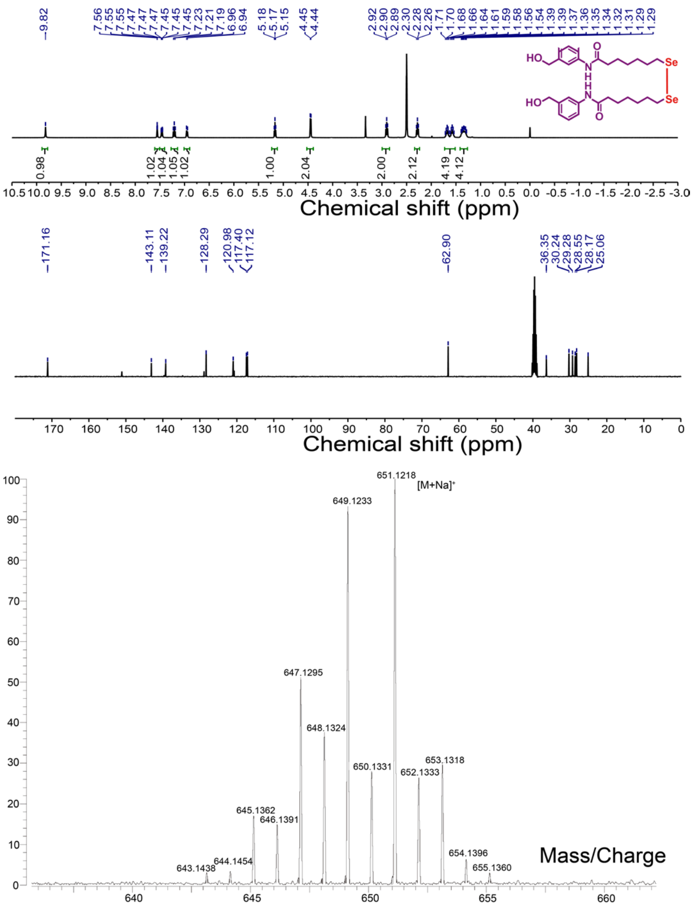

Figure S3. Structure confirmation of SeSAHA in DMSO-d_6. (A): ^{1}H NMR spectrum of SeSAHA (400 MHz, DMSO-d_6). (B): ^{13}C NMR spectrum of SeSAHA (101 MHz, DMSO-d_6). (C): MS spectrum of SeSAHA.

A
B

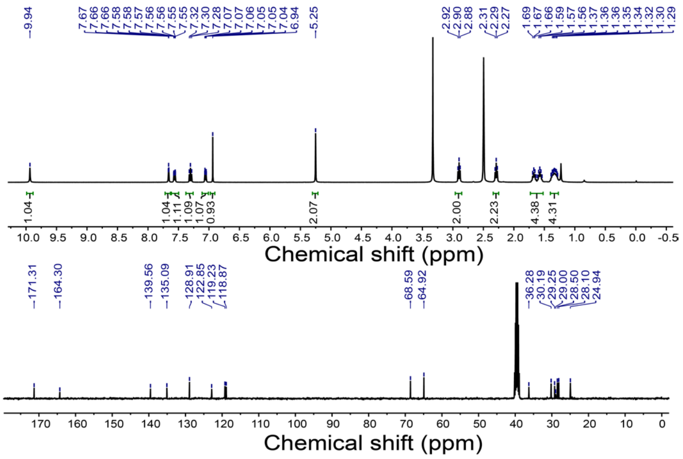

Figure S4. Structure confirmation of SeSA-DCA in DMSO-d_6. (A): ^{1}H NMR spectrum of SeSA-DCA (400 MHz, DMSO-d_6). (B): ^{13}C NMR spectrum of SeSA-DCA (101 MHz, DMSO-d_6).

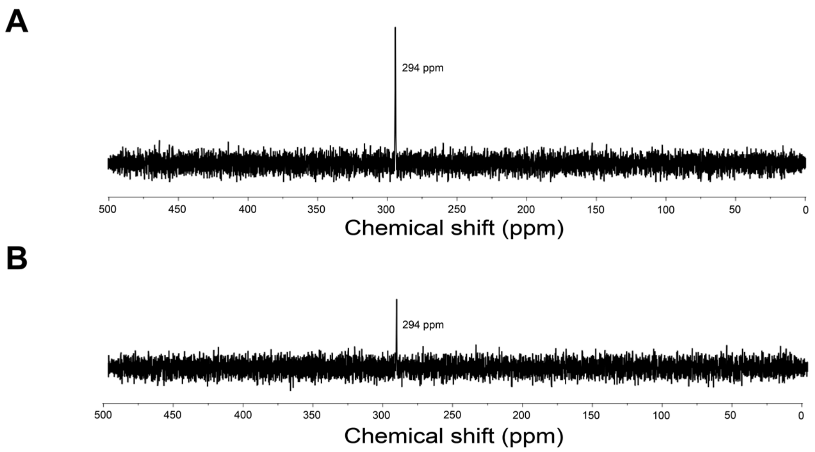

Figure S5. ^{77}Se NMR spectrum of (A) SeSAHA and (B) SeSA-DCA in DMSO-d_6.

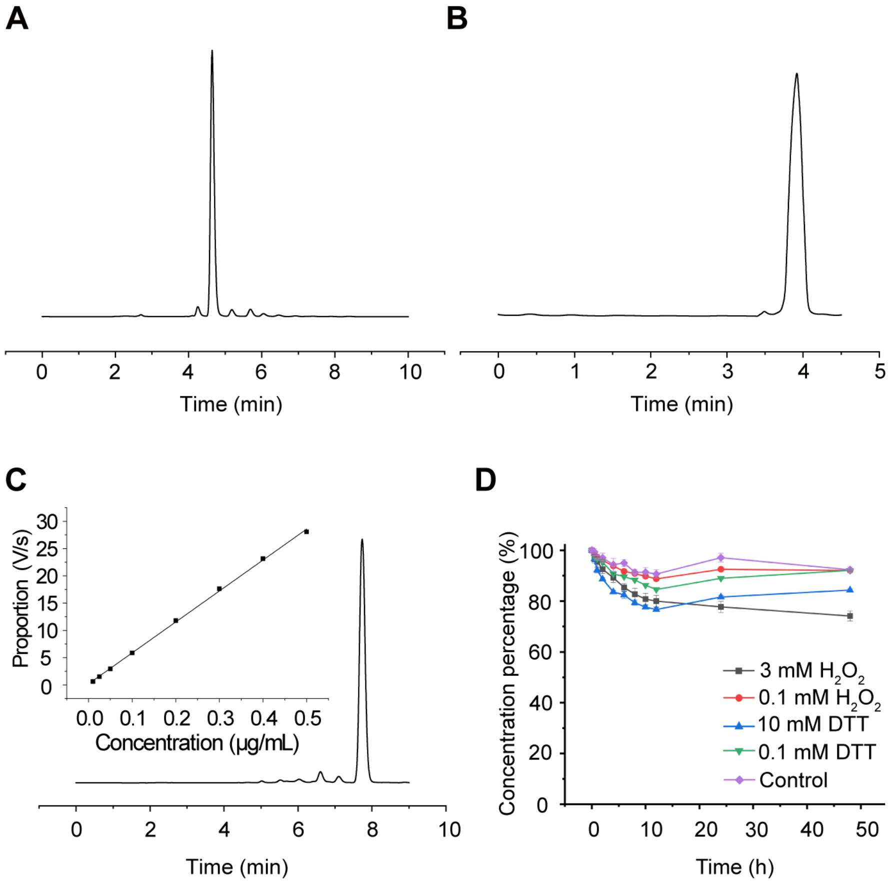

Figure S6. The redox response of SeSA-DCA was determined by HPLC. Schematic diagram of HPLC peak positions of SeSAHA (A) and DCA (B). (C) Schematic diagram of HPLC peak position and standard curve of SeSA-DCA ($y = 5.67 \times 10^7x + 193812$, $R^2 = 0.9991$). (D) Line graph of the decomposition ratio of SeSA-DCA oxidation-reduction (n=3).

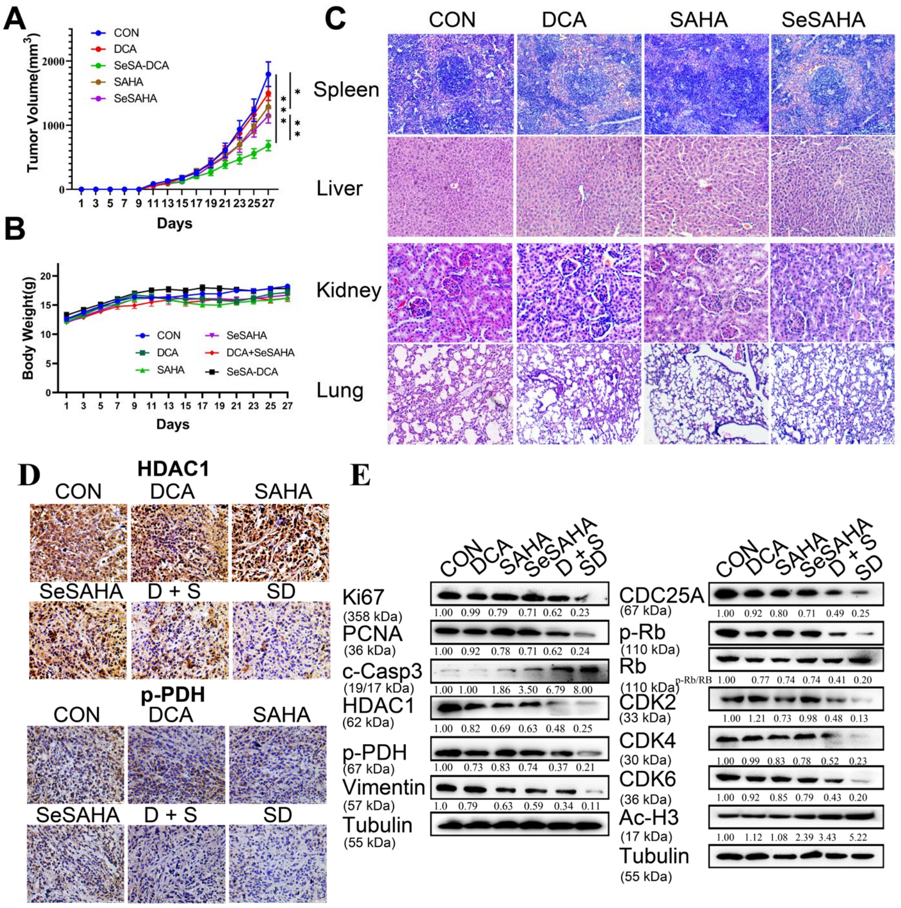

Figure S7. SeSA-DCA inhibits the growth of transplanted tumor and its mechanism. (A): Tumor growth curve of PC3 tumor-bearing mice in response to different treatments. (B)：Effects of different treatments on body weight growth curve of PC3 tumor-bearing mice. (C) HE staining of spleen, liver, kidney, and lung. (D) Immunohistochemical study on the changes of HDAC1, p-PDH protein in PC3 xenografts. (E) Western blotting confirmed the changes of the cell cycle, proliferation, apoptosis, metastasis, and other related proteins in PC3 transplanted tumor. *$p < 0.05$, **$p < 0.01$, ***$p < 0.001$. D + S: DCA + SeSAHA; SD: SeSA-DCA.

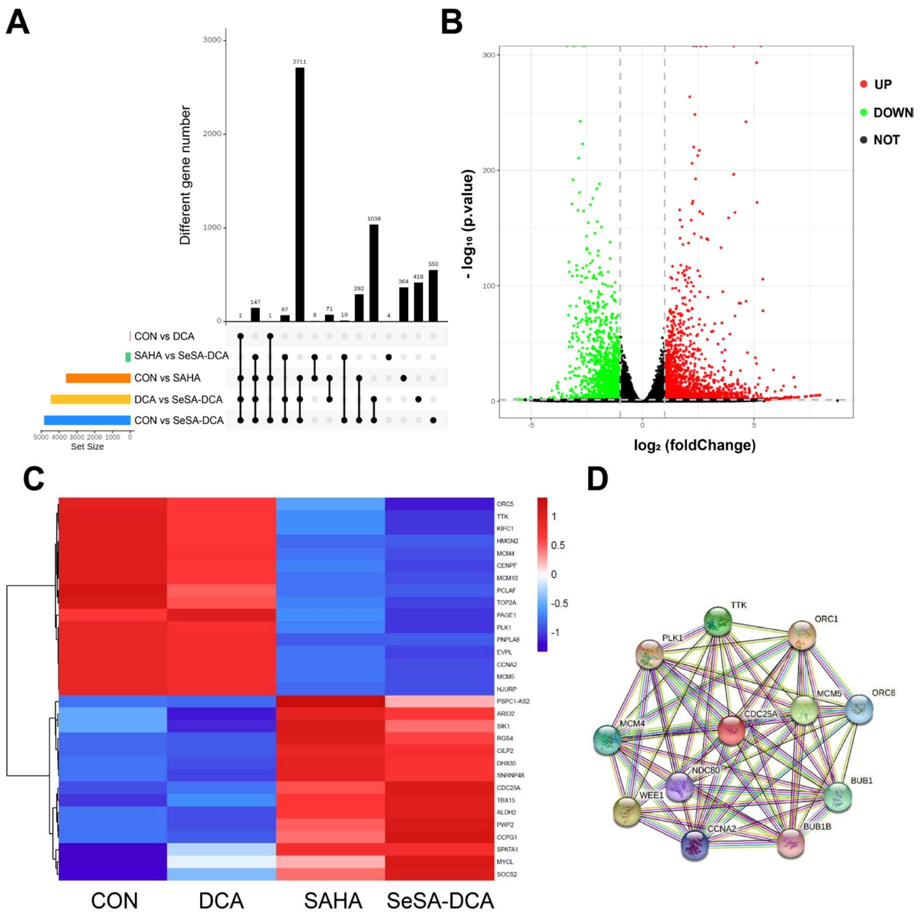

Figure S8. Selection of potential targets for SeSA-DCA. (A): Venn diagram of genetic changes in PC3 cells treated with drugs. (B): Volcanic diagram of genetic changes of SeSA-DCA compared with CON in PC3 cells. (C) Changes of cell cycle related genes in PC3 cells treated with drugs. (D) The STRING database shows the relationship between related molecules in the cell cycle pathway.

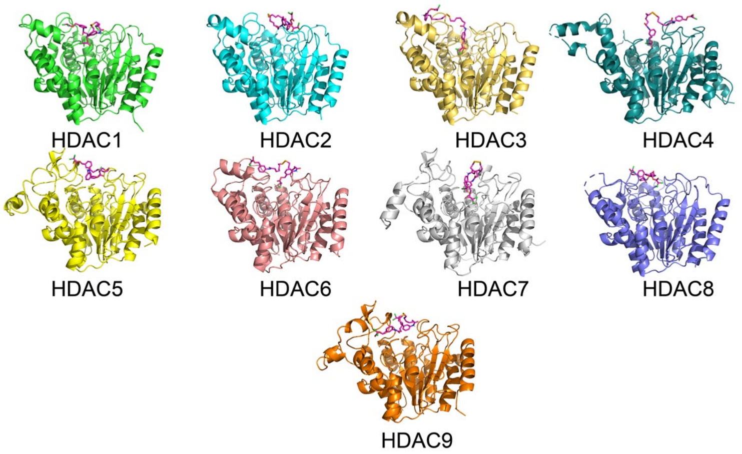

Figure S9. Molecular docking results of SeSA-DCA and HDACs.

Table S1. Cell lines dose: Unit/μM

Cell lines	DCA	SAHA	SeSAHA	DCA + SeSAHA	SeSA-DCA
PC3	0.02	0.02	0.02	0.02 + 0.02	0.02
DU145	0.5	0.5	0.5	0.5 + 0.5	0.5

For human genes:

Table S2. The primer sequences were used for real-time PCR assay.

Target	Forward primer（5′-3′）	Reverse primer（5′-3′）
CDC25A	GCCACTTTGTCTGATGAGG	CAGCTTGCATCGGTTGT
GAPDH	CCTTCCGTGTCCCCACT	GCCTGCTTCACCACCTTC
